# High Prevalence of *SOD1* Pathogenic Variants in the UK Biobank: Implications for Early Intervention in Amyotrophic Lateral Sclerosis

**DOI:** 10.1002/ana.78195

**Published:** 2026-03-19

**Authors:** Delia Gagliardi, Chiara Villella, Matteo Zanovello, Virginia Iacobelli, Stefania Corti, Giacomo Pietro Comi, Pietro Fratta, Henry Houlden, Arianna Tucci, Dario Ronchi

**Affiliations:** ^1^ Fondazione IRCCS Ca' Granda Ospedale Maggiore Policlinico, Neurology Unit Milan Italy; ^2^ Dino Ferrari Center, Department of Pathophysiology and Transplantation University of Milan Milan Italy; ^3^ Department of Neuromuscular Diseases, UCL Queen Square Institute of Neurology University College London London UK; ^4^ Fondazione IRCCS Ca' Granda Ospedale Maggiore Policlinico, Neuromuscular and Rare Disease Unit Milan Italy; ^5^ The Francis Crick Institute London UK; ^6^ Neurogenetics Unit, National Hospital for Neurology and Neurosurgery London UK; ^7^ William Harvey Research Institute Queen Mary University of London London UK; ^8^ Department of Neurodegenerative Disease UCL Queen Square Institute of Neurology, UCL London UK

## Abstract

**Objective:**

*SOD1* is the second most frequently mutated gene in European patients with amyotrophic lateral sclerosis (ALS). Given the recent authorization of *SOD1*‐targeted antisense oligonucleotides for *SOD1*‐ALS, prompt screening for *SOD1* mutations in patients with ALS patients is highly recommended. Large‐scale genomic analysis could inform on the population‐based prevalence of *SOD1* mutation carriers, who would potentially benefit from treatment. We aim to determine the number of people with pathogenic *SOD1* variants in the UK Biobank (UKB), to address a critical gap between clinical and genetic prevalence of *SOD1*‐ALS.

**Methods:**

We analyzed *SOD1* variants within exome sequencing data from 470,000 individuals aged over 40 years. Pathogenicity was evaluated using referenced databases and American College of Medical Genetics and Genomics (ACMG) guidelines. Leveraging the UKB carrier frequency and age at onset data, we estimated the genetic prevalence of *SOD1*‐ALS. We examined factors that may influence penetrance.

**Results:**

We identified 122 individuals with monoallelic *SOD1* coding variants, 93.4% of whom were asymptomatic. Additionally, the low‐penetrance p.Asp91Ala variant was observed in heterozygosis in 535 subjects, whereas it was never found in homozygosis. Excluding this variant, the expected number of people developing *SOD1*‐ALS is 1.04:100,000 in the UK population, 4 times higher than clinically reported figures. Symptomatic carriers had significantly increased levels of serum neurofilament at baseline. Age‐related penetrance was higher in non‐p.Asp91Ala carriers versus p.Asp91Ala carriers. Long‐term survivor status was associated with p.Asp91Ala genotype, older age, and lower neurofilament levels.

**Interpretation:**

Incomplete and age‐related penetrance, along with underascertainment due to disease heterogeneity and limitations in data collection, likely account for the reduced number of symptomatic patients identified. Our findings highlight the need to identify genetic and environmental factors, as well as biological indicators, able to influence disease penetrance and phenoconversion risk in presymptomatic carriers and to predict treatment response in patients. ANN NEUROL 2026;99:1502–1515

In 1993, pathogenic variants in the superoxide dismutase 1 (*SOD1*) gene were identified as the first genetic cause of amyotrophic lateral sclerosis (ALS), marking a pivotal moment in understanding the disease's molecular basis.^1^ This discovery initiated a 30‐year journey from gene identification to the development of targeted gene therapies, creating a pathway toward potential disease prevention and highlighting the critical importance of identifying at‐risk individuals before symptom onset.

*SOD1*‐ALS accounts for approximately 1 to 6% of ALS cases globally, with significant geographic variation in prevalence, representing approximately 1 to 2% of sporadic cases (sALS) and up to 20% of familial forms (fALS).^2^ Clinical presentations of *SOD1*‐ALS are remarkably heterogeneous, with a median age of onset approximately 47 to 48 years, variable disease progression rates, and predominantly spinal rather than bulbar onset of symptoms.^3^ Penetrance of *SOD1*‐ALS is incomplete and varies across different mutations,^4^ ranging from almost full penetrance in the aggressive p.Ala5Val variant to a polymorphic frequency in some populations for the heterozygous p.Asp91Ala. A recent study estimated a penetrance for *SOD1*‐ALS of 54% at a population level.^5^

Over 234 different variants, unevenly distributed across the 5 exons of *SOD1*, have been reported.^6^ Despite efforts in assessing the pathogenicity of *SOD1* variants,^7^ there are no consensus criteria to definitely prove their detrimental role. The vast majority of them are missense variants; however, more than 30 nonsense pathogenic variants have been reported.^4^ Pathogenic variants are usually heterozygous; however, for some variants (p.Ser69Pro, p.Leu85Phe, p.Asn87Ser, p.Asp91Ala, p.Leu118Val, p.Val120Leu, p.Leu127Ser, p.Leu145Ser, and p.Gly28delGGACCA), a recessive inheritance has been reported.^8^, ^9^, ^10^

Biallelic variants in p.Asp91Ala, along with compound heterozygosity between the p.Asp91Ala monoallelic variant and other *SOD1* variants, are associated with fALS in Scandinavia. Despite being described in a heterozygous state in some patients with ALS,^3^ the pathogenic role of the p.Asp91Ala variant is still debated. The absence of co‐segregation with the heterozygous p.Asp91Ala carrier in ALS pedigrees, the normal enzymatic activity, and the presence of only minimal *SOD1* microaggregates in postmortem brains, similarly to those observed in other neurodegenerative disorders, question the pathogenicity of the heterozygous p.Asp91Ala variant.^11^

Recent therapeutic advances have targeted the toxic gain‐of‐function mechanism through gene silencing approaches. Tofersen, an antisense oligonucleotide that reduces SOD1 protein expression by inducing RNase H‐mediated degradation of *SOD1* messenger RNA, received accelerated approval from the US Food and Drug Administration (FDA) based on its ability to reduce plasma neurofilament light chain (NfL) concentrations, a biomarker reasonably likely to predict clinical benefit.^12^ Moreover, longitudinal follow‐up of pre‐symptomatic *SOD1* variant carriers has revealed that plasma NfL concentrations rise 6 to 12 months before phenoconversion to clinically manifest ALS.^13^ This finding has enabled the design of preventive trials (ATLAS, NCT04856982), which aim to prevent or delay the onset of clinical symptoms by administering tofersen when NfL levels rise above a predefined threshold in pre‐symptomatic carriers of highly penetrant *SOD1* variants.

*SOD1*‐ALS is currently the only genetic ALS form for which a molecular therapy is available, and a clinical study on presymptomatic individuals carrying aggressive variants is ongoing.^13^ Moreover, *SOD1*‐ALS clinical manifestation can be subtle and slowly progressing, resulting in a substantial delay (months or even years) in diagnosis.^14^ Thus, identifying potential carriers in the general population represents an unprecedented opportunity to intervene before irreversible neurodegeneration occurs, significantly altering the disease trajectory for affected individuals.

Our study aims to identify carriers of pathogenic and likely pathogenic *SOD1* variants in a large population dataset, in an era where a molecular therapy for *SOD1*‐ALS is available and is currently being investigated in a clinical trial involving presymptomatic individuals. In addition, we aim to estimate the genetic prevalence of *SOD1*‐ALS in the UK population, potentially expanding our understanding of *SOD1*‐ALS epidemiology and informing current and future preventive and therapeutic strategies.

## Methods

### Ethics Approval

All research activities followed protocols approved by the Research Ethics Committee (UKBB reference: 16/NW/0274), with all study participants providing informed consent.

### Study Population and Variant Detection

The UK Biobank (UKB) cohort is a large repository including longitudinal demographic, phenotypic, and genomic data from more than 500,000 individuals between the ages of 40 and 69 years, recruited across the United Kingdom between 2006 and 2010.^15^ Leveraging whole‐exome sequencing (WES) data from 470,000 participants, we extracted *SOD1* coding variants with a minor allele frequency lower than 0.001. Variant annotation was performed using the Annovar tool,^16^ and synonymous, splicing, and non‐coding variants (in the 3′ and 5′ UTR) were excluded. Variants were classified as pathogenic, likely pathogenic, or of uncertain significance following American College of Medical Genetics and Genomics (ACMG) guidelines,^17^ and according to reference databases specific to ALS, including ALSOD, SODCOD, and Project Mine.^6^, ^18^, ^19^ We established a consensus classification by integrating evidence from multiple sources, with priority given to functionally validated variants with established genotype–phenotype correlations.

### Prevalence Estimation Methodology

To estimate the number of people affected by *SOD1*‐ALS in the UK population, we used a previously described modeling approach,^20^ which incorporated:

1. *SOD1* carrier frequency in the UKB cohort, calculated by dividing the number of exomes with a pathogenic or likely pathogenic *SOD1* variant by the total number of sequences (n = 470,000). Confidence intervals (CIs) at 95% were calculated as previously described.^20^
2. Age‐stratified population statistics from the UK Office of National Statistics.^21^
3. Distribution of age at onset of *SOD1*‐ALS, derived from a cohort of 1,122 patients.^12^ Because the p.Ala5Val variant was not identified in the UKB cohort, we excluded patients harboring this variant from the age‐at‐onset distribution analysis to ensure our model accurately reflected the variant spectrum observed in our dataset.
4. Disease penetrance of *SOD1*‐ALS, which was recently estimated to be 54% at a population level.^5^ Additionally, we calculated genetic prevalence estimates assuming penetrance values ranging from 30 to 100%.
5. Median disease survival, to adjust for mortality effects. Total *SOD1*‐ALS median survival is reported to be 2.7 years^12^; however, because forms with the p.Ala5Val variants have a more aggressive disease course (survival = 1.4 years), we considered the disease survival for non‐p.Ala5Val *SOD1*‐ALS as more representative (median survival = 6.8 years).^12^

After checking relatedness (see “Phenotypic analysis” below), to avoid overestimation of carrier frequency, we counted carriers from the same family as a single carrier. Heterozygous p.Asp91Ala has been reported as a low‐penetrance variant, and its pathogenic effect in *SOD1*‐ALS is still debated. This variant appears to cause disease primarily in the homozygous state or in compound heterozygosity with other pathogenic variants, with limited evidence for pathogenicity in heterozygous carriers.^11^ For this reason, we performed 2 different estimates of *SOD1*‐ALS genetic prevalence, with and without the p.Asp91Ala variant (Supplementary Tables S2 and S3), to provide a comprehensive range of potential disease burden.

The new genetic estimate was compared to the clinical figure derived from literature data. Considering that *SOD1* mutations account for up to 20% of fALS, which represents 10% of all ALS cases, and 1 to 2% of sALS, which constitutes 90% of ALS cases, and given an ALS prevalence of 8 per 100,000 (range = 5–12 per 100,000),^22^ the prevalence of *SOD1*‐ALS based on clinical observation would be 0.27 per 100,000.

### Phenotypic Analysis

Age at recruitment, gender, and ethnicity were collected from UKB baseline assessment data. We checked the presence of relatives using the “Genetically relatedness pairing” variable. The strength of the inferred relationship was assessed through the “Genetic relatedness factor” or kinship coefficient, that is, the probability that 2 random alleles at the same locus are identical by descent, and the “Genetic relatedness IBS0,” which measures the proportion of single‐nucleotide polymorphisms (SNPs) where 2 individuals share no alleles. To investigate potential disease manifestations among *SOD1* variant carriers, we implemented a comprehensive phenotyping approach using multiple data sources. We extracted medical diagnosis codes, using International Classification of Diseases, 10th Revision (ICD‐10) from linked health care records and self‐reported health conditions from participant questionnaires, along with the reported cause of death for deceased patients. We developed a hierarchical classification system to categorize symptom severity, where subjects with at least one code referred to the condition and/or to motor neuron degeneration signs and symptoms (see Supplementary Table S3) were considered symptomatic. Symptoms were further stratified into strong, moderate, and weak evidence categories based on the specificity of ICD‐10 codes to motor neuron disease (MND) and known *SOD1*‐ALS manifestations. Participants were classified as symptomatic only if they had ICD‐10 codes for MND corresponding to strong or moderate diagnostic certainty. Those with weak diagnostic codes or no MND‐related codes were classified as asymptomatic. Plasma NfL normalized values were extracted from Olink proteomic data. Only measurements from the initial assessment visit (2006–2010) were used for analysis, given the low number of available measurements for follow‐up visits.

### Penetrance Estimation Analysis

We defined “long‐term survivors” (LTS) as participants aged ≥70 years who remained asymptomatic (or weakly symptomatic) and alive at the time of analysis. This age threshold represents the 95th percentile of typical ALS onset age in the literature.^12^

Penetrance for p.Asp91Ala, non‐p.Asp91Ala, and each *SOD1* variant was calculated as the proportion of symptomatic individuals among those at risk. Baseline NfL values were summarized using medians, with sample size weighting. Penetrance analysis was performed using Kaplan–Meier survival analysis. The event of interest was the development of symptomatic MND, with time‐to‐event defined as current age for asymptomatic participants or age at diagnosis for symptomatic participants. Participants who died without developing symptoms were censored at age of death. Penetrance was calculated as 1 minus the survival probability at specific ages (60, 70, and 80 years). Differences in penetrance between p.Asp91Ala and non‐p.Asp91Ala genotypes were assessed using the log rank test. CIs for penetrance estimates were derived from the Kaplan–Meier survival curves. Time‐to‐symptom onset analysis was performed using Cox proportional hazards regression.

### Statistical Analysis

Continuous variables were compared between groups using the Mann–Whitney *U* test for 2 groups or the Kruskal–Wallis test for multiple groups, given non‐normal distributions. Categorical variables were compared between the same groups using Fisher's exact test. Survival curves were compared using the log rank test. Multivariable logistic regression analysis was performed to identify predictors of long‐term survivor status in *SOD1* carriers. The primary model included age, sex, genotype, and NfL levels as covariates. Odds ratios (ORs) with 95% CIs were calculated for all predictors.

All data were analyzed using R Studio version 4.5.1, and the statistical significance threshold was set to *p <* 0.05. Plots were generated using the ggplot2 package in R software. Graphs representing proportions of symptomatic and asymptomatic individuals were generated using GraphPad Prism version 10.

## Results

**FIGURE 1:**
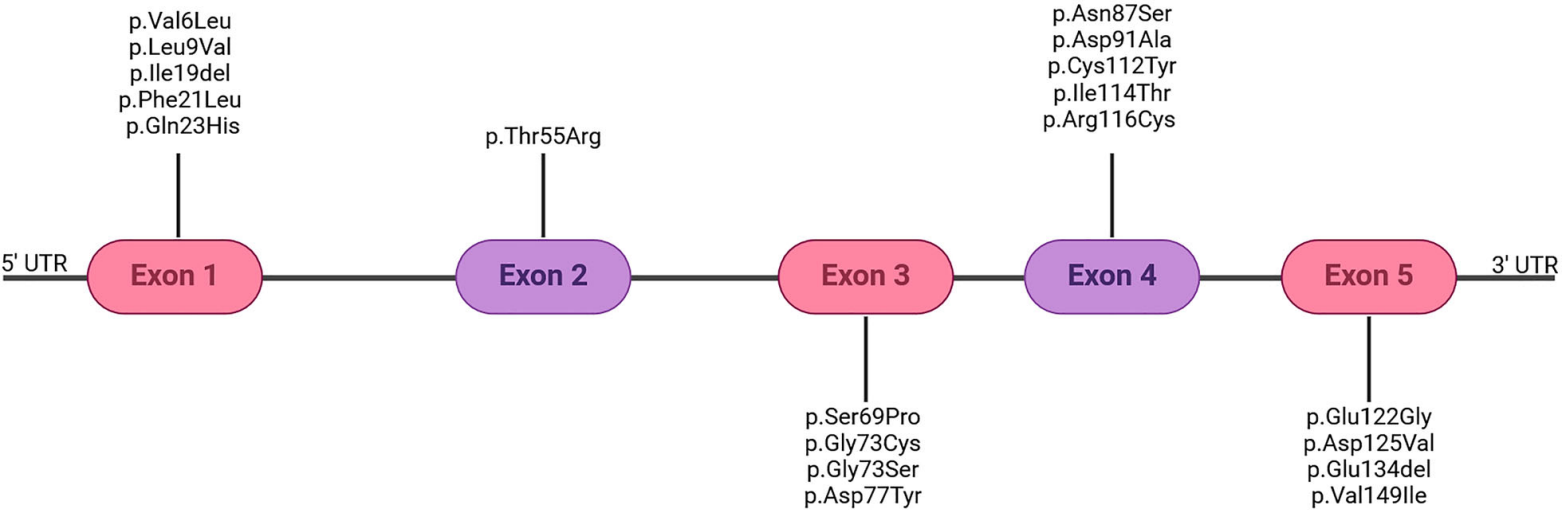
Schematic representation of the *SOD1* gene showing the location of 20 distinct coding variants identified across 657 patients. Exons are depicted as colored boxes, and variants are marked along the gene structure according to their position. [Color figure can be viewed at www.annalsofneurology.org]

We identified a total of 19 coding variants previously reported in the scientific literature and currently classified as pathogenic (N = 9) and likely pathogenic (N = 10) across 122 participants in the UKB cohort (Fig 1 and Table 1a and 1b). Among them, 2 individuals carrying the p.Ile19del variant were related (IBSO 0, kinship coefficient 0.25). Additionally, 535 individuals were found to carry the low‐penetrance heterozygous p.Asp91Ala variant; 2 of them belonging to the same family (IBSO 0, kinship coefficient 0.0223). Notably, no coding homozygous *SOD1* variants were identified in the cohort. None of these individuals harbored other *SOD1* variants.

**TABLE 1a:** *SOD1* Pathogenic and Likely Pathogenic Variants Identified in the UK Biobank Cohort

Nucleotide change	Protein change	Exon	No. of carriers	ACMG criteria	P/LP	Databases	Reported ancestry	Citations
c.341T>C	p.Ile114Thr	4	32	PS4, PS3, PM1, PP2, PM2, PM5, PP3	P	ALSOD; ProjectMINE: Moderate	British (32)	PMID: 18428003
c.69G>C	p.Gln23His	1	25	PM1, PP2, PM2, PM5, PP3, PP5	LP	ALSOD, SODCOD	British (25)	PMID: 21603025
c.217G>T	p.Gly73Cys	3	25	PM1, PP2, PM2, PM5, PP3	LP	ALSOD, SODCOD, ProjectMINE: Moderate	British (23), Any other white background (1), Prefer not to say (1)	PMID: 16435343; PMID: 1842803
c.374A>T	p.Asp125Val	5	6	PS4, PS3, PM1, PP2, PM2, PM5, PP3	P	ALSOD, SODCOD	British (6)	PMID: 8938700; PMID: 11854284
c.217G>A	p.Gly73Ser	3	5	PS4, PS3, PM1, PP2, PM2, PP3	P	ALSOD, SODCOD	British (5)	PMID: 9506558; PMID: 21084099; PMID: 11854284
c.25C>G	p.Leu9Val	1	4	PM1, PP2, PM2, PM5, PP3	LP	ALSOD, SODCOD, ProjectMINE: Moderate	British (4)	PMID: 14506936; PMID: 28430856; PMID: 14506936; PMID: 24908169
c.164C>G	p.Thr55Arg	2	4	PM1, PP2, PM2, PM5, PP3	LP	ALSOD, SODCOD	British (3), Any other white background (1)	PMID: 14506936
c.346C>T	p.Arg116Cys	4	4	PS4, PM1, PP2, PM2, PM5, PP3	P	ALSOD	British (2), Indian (1), Other ethnic group (1)	PMID: 23182243
c.16G>C	p.Val6Leu	1	3	PM1, PP2, PM2, PP3	LP	ALSOD, SODCOD	British (3)	PMID: 21700728
c.229G>T	p.Asp77Tyr	3	3	PS4, PS3, PM1, PP2, PM2, PM5, PP3	P	ALSOD, SODCOD, ProjectMINE: Moderate	British (3)	PMID: 9365366; PMID: 18428003; PMID: 28430856

Abbreviations: ACMG, American College of Medical Genetics and Genomics; ALSOD, ALS Online Database; LP, likely pathogenic; P, pathogenic. ProjectMINE: population‐scale ALS whole‐genome sequencing project; PM, pathogenic moderate; PP: pathogenic supporting; PP5, reputable source reports variant as pathogenic; PS, pathogenic strong; PS1, same amino acid change as established pathogenic variant; SODCOD, SOD1 database.

**TABLE 1b:** *SOD1* pathogenic and likely pathogenic variants identified in the UK Biobank cohort (continued).

Nucleotide change	Protein change	Exon	No. of carriers	ACMG criteria	P/LP	Databases	Reported ancestry	Citations
c.51_53del	p.Ile19del	1	2	PM2, PM4, PM1	LP	ALSOD	British (2)	PMID: 24296360
c.445G>A	p.Val149Ile	5	2	PS4, PM1, PP2, PM2, PM5, PP3	P	ALSOD	British (2)	PMID: 8815157; PMID: 19483195
c.397_399del	p.Glu134del	5	1	PM4, PM5, PM2, PM1	LP	ALSOD, SODCOD, ProjectMINE: Moderate	British (1)	PMID: 893870; PMID: 11854284
c.63C>G	p.Phe21Leu	1	1	PS4, PM1, PP2, PM2, PM5, PP3	P	ALSOD	Not available	PMID: 22722621; PMID: 35260199
c.69G>T	p.Gln23His	1	1	PM5, PS1, PM1, PP2, PM2, PP3	LP	ALSOD	British (1)	PMID: 21603026
c.205T>C	p.Ser69Pro	3	1	PS4, PM1, PP2, PM2	LP	/	British (1)	PMID: 36472686
c.260A>G	p.Asn87Ser	4	1	PS4, PM1, PP2, PM2, PP3	P	ALSOD, SODCOD, ProjectMINE: Moderate	Indian (1)	PMID: 20577002; PMID: 30637102; PMID: 9556377
c.335G>A	p.Cys112Tyr	4	1	PS4, PS3, PM1, PP2, PM2, PP3	P	ALSOD, SODCOD	British (1)	PMID: 18428003; PMID: 20888599; PMID: 28430856
c.365A>G	p.Glu122Gly	5	1	PM1, PP2, PM2, PP3	LP	SODCOD	Other ethnic group (1)	PMID: 25336041
c.272A>C	p.Asp91Ala	4	535	PM1, PP2, PM2, PM5, PP5	LP	ALSOD, SODCOD	British (499), Any other white background (19), Irish (5), Indian (5), Pakistani (5), White and Black Caribbean (1), Other ethnic group (1)	PMID: 10439968

Abbreviations: ACMG, American College of Medical Genetics and Genomics; ALSOD, ALS Online Database; LP, likely pathogenic. P, pathogenic. ProjectMINE, population‐scale ALS whole‐genome sequencing project; PM, pathogenic moderate; PP: pathogenic supporting; PP5, reputable source reports variant as pathogenic; PS, pathogenic strong; PS1, same amino acid change as established pathogenic variant; SODCOD, SOD1 database.

Finally, 66 additional previously unreported variants were identified in 351 individuals. Among them, 36 were pathogenic or likely pathogenic, whereas the remaining were classified as variants of unknown significance. Considering the absence of clinical or functional evidence of pathogenicity, we excluded them from further analyses (Supplementary Table S4).

Demographic analysis of the 657 identified *SOD1* carriers of pathogenic and likely pathogenic variants revealed a mean age at recruitment of 58 years (range = 50.5–63 years), with 45.8% being women (Table 2). The majority (N = 590, 89.8%) were Caucasian.

**TABLE 2:** *SOD1* Carriers Including the p.Asp91Ala Variant

	SOD1 Carriers (*N* = 657)	Symptomatic SOD1 Carriers (*N* = 17)	Asymptomatic SOD1 Carriers (*N* = 640)	*p* value
Age at recruitment, yr				
Median [IQR]	58 [50.5 to 63]	62 [57.5 to 64.5]	57 [50 to 63]	0.011
Gender, *N* (%)				
M	356 (54.2)	6 (35.3)	350 (54.7)	0.141
F	301 (45.8)	11 (64.7)	290 (45.3)	
Genotype, *N* (%)				
Non‐p.Asp91Ala	122 (18.6)	8 (47.1)	114 (17.8)	0.006
p.Asp91Ala	535 (81.4)	9 (52.9)	526 (82.1)	
Baseline NfL (normalized)				
Median [IQR]	−0.01 [0.28 to 0.48]	6.13 [0.87 to –16.56]	−0.06 [−0.29 to –0.45]	0.002
Reported ethnicity, *N* (%)				
European	613 (93.3)	15 (88.2)	598 (93.4)	
Irish	5 (0.7)	–	5 (0.8)	>0.999
Other White background	21 (3.2)	2 (11.8)	19 (3)	
Indian	7 (1.1)	–	7 (1.1)	
Pakistani	5 (0.7)	–	5 (0.8)	
White and Black Caribbean	1 (0.1)	–	1 (0.1)	
Other ethnic group	4 (0.6)	–	3 (0.5)	
Not available	2 (0.3)	–	2 (0.3)	

*Note*: Continuous variables are expressed as median and [interquartile range]. Categorical variables are expressed as count and percentages.

Abbreviations: IQR, interquartile range; NfL, neurofilament light chain.

**FIGURE 2:**
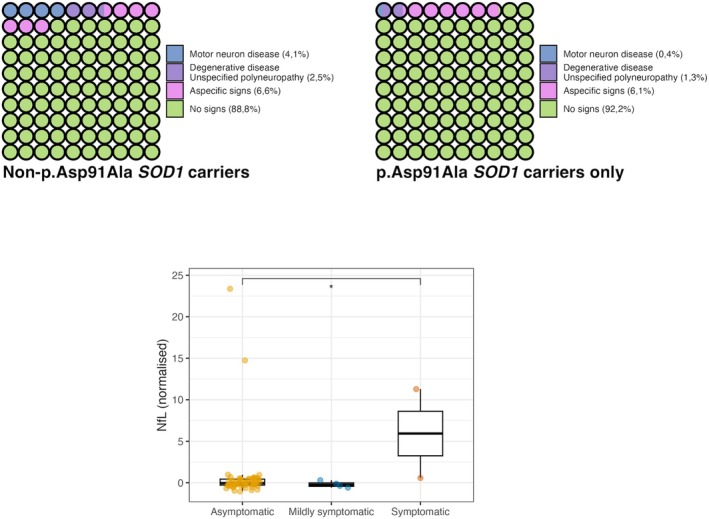
*SOD1* carriers according to symptomatic state. (A, B). Waffle plots representing the proportion of symptomatic patients among *SOD1* non‐p.Asp91Ala carriers (A) and in p.Asp91Ala carriers only (B). Bar plot representing normalized serum NfL levels at baseline in *SOD1* carriers grouped by symptomatic status (symptomatic, weak symptoms, and asymptomatic). [Color figure can be viewed at www.annalsofneurology.org]

Clinical phenotyping identified 17 symptomatic individuals (2.6%) in the overall cohort. Definite MND diagnosis (strong evidence) was observed in 7 (1.1%) individuals (4.1% of the non‐p.Asp91Ala carriers and only 0.4% among the p.Asp91Ala carriers), whereas nonspecific neurodegenerative conditions or polyneuropathy (moderate evidence) were detected in 10 (1.5%) individuals (N = 3, 2.5% of non‐p.Asp91Ala and N = 7, 1.3% of p.Asp91Ala carriers). Nonspecific neurological symptoms (weak evidence) were detected in 40 individuals, 6.1% of subjects (Fig 2A, B). Among the 657 participants, 600 (91.3%) were alive at the time of analysis (current age = 74 years [range = 67–79]; Supplementary Table S5). For subsequent analyses, only carriers with strong or moderate evidence of symptoms were considered symptomatic.

Comparing symptomatic versus asymptomatic carriers, a significant difference was found in age at recruitment (62 [range = 57.5–64.5] vs. 57 [range = 50–63] years, *p* = 0.011), which was still present when considering only the non‐p.Asp91Ala carriers, but no significant differences in sex or reported ancestry distribution were observed. Baseline plasma NfL levels (available for a subset of our cohort, N = 74) were significantly increased in symptomatic versus asymptomatic individuals (*p* = 0.002) and in symptomatic versus carriers with weak symptoms (*p* = 0.009; Fig 2C). Moreover, the association between genotype and symptomatic status was statistically significant (*p* = 0.006), with non‐p.Asp91Ala carriers having substantially higher odds of being symptomatic compared with p.Asp91Ala carriers.

The carrier frequency for pathogenic and likely pathogenic *SOD1* variants in the UKB was calculated as 1 out of 3,884 individuals (95% CI = 1 in 3,251 to 1 in 4,681). When including the heterozygous p.Asp91Ala variant, the carrier frequency increased substantially to 1 in 718 individuals (95% CI = 1 in 666 to 1 in 777 1 in 677 to 1 in 755). The carrier frequency of the heterozygous p.Asp91Ala variant in the UKB is 1 in 880 (95% CI = 1 in 809 to 1 in 860). We obtained similar carrier frequency when considering Caucasians only.

**FIGURE 3:**
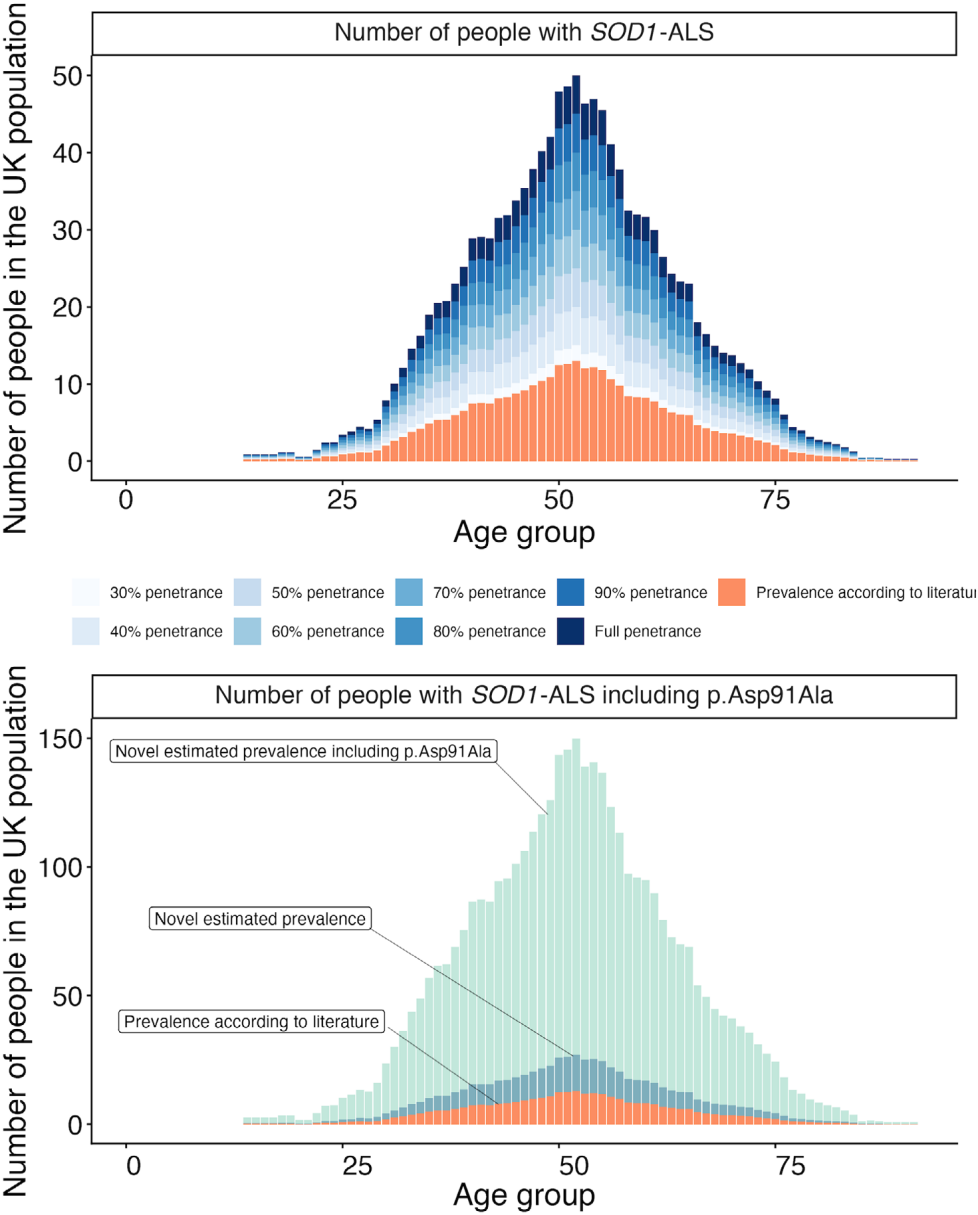
Estimated number of people with *SOD1*‐ALS due to pathogenic and likely pathogenic variants in the UK Biobank cohort. (A) Prevalence estimates (area with different *shades of blue*) are calculated using a carrier frequency of 1 in 3,884 and do not include carriers of the p.Asp91Ala variant, and are compared to the clinical prevalence from the literature (*orange area*). Using penetrance of 30%, 40%, 50%, 60%, 70%, 80%, 90%, and 100%, prevalence estimates are 0.58 = 100,000; 0.77 = 100,000; 0.96 = 100,000; 1.16 = 100,000; 1.35 = 100,000; 1.54 = 100,000; 1:73 = 100,000; and 1.93 = 100,000, respectively. (**B**) Novel estimated prevalence from non‐p.Asp91Ala carriers (*dark blue area*) is calculated using a carrier frequency of 1 in 3,884 and is compared to the estimated prevalence from all *SOD1* carriers, including the p.Asp91Ala carriers (carrier frequency 1/718, *light blue area*) and to the clinical prevalence from the literature (*orange area*). The UK population count by age is multiplied by the disease allele frequency of *SOD1* variants and the age of onset distribution of *SOD1*‐ALS, and corrected for median survival. Penetrance is assumed to be 54%. X‐axis = the age bins are 5 years each; y‐axis = estimated number of affected individuals. [Color figure can be viewed at www.annalsofneurology.org]

To estimate the prevalence of *SOD1*‐ALS in the UK population, we applied a disease modeling approach using age‐at‐onset data from 598 patients with *SOD1*‐ALS, excluding the p.Ala5Val variant.^12^ Under varying penetrance assumptions (30% to 100%), we estimated the genetic prevalence of *SOD1*‐ALS to be 0.58 to 1.93 per 100,000 individuals (Fig 3A). Using 54% as disease penetrance, the number of people with *SOD1*‐ALS was estimated at 1.04 cases per 100,000 individuals, which is approximately 4 times higher than the prevalence derived from clinical observation (0.27 per 100,000). When including the low‐penetrance heterozygous p.Asp91Ala variant and using age‐at‐onset data from 667 patients, the prevalence of *SOD1*‐ALS increased to 5.63 cases per 100,000 individuals (Fig 3B).

In order to explain the gap between estimated prevalence and clinical prevalence, we performed a cross‐sectional analysis of potential modifiers of disease penetrance in *SOD1*‐ALS, including genotype, age, sex, and plasma NfL levels (available for a subset of our cohort, N = 74). The lack of longitudinal follow‐up for most NfL measurements prevented a longitudinal investigation.

Considering symptomatic carriers only, no difference in terms of current age, age at recruitment, sex, and plasma NfL was found between the p.Asp91Ala and non‐p.Asp91Ala genotypes.

Among participants aged 70 years and above who remained alive and asymptomatic, we identified 389 LTS. The majority were p.Asp91Ala carriers (n = 321, 60% of all p.Asp91Ala carriers) compared with non‐p.Asp91Ala carriers (n = 68, 12.7% of all non‐p.Asp91Ala carriers). Comparing LTS with symptomatic carriers (n = 17), we found a significant difference between current age (78 [range = 74–81] vs. 78 [range = 68.8–78.3] years, *p* = 0.025), p.Asp91Ala genotype (n = 321, 82.5% of LTS vs. 9, 52.9% of symptomatic, *p* = 0.006) and baseline NfL levels (0.2 [range = −0.2 to –0.5] vs. 6.1 [range = 0.9–16.6], *p* < 0.001). Age at recruitment and sex did not show any significant difference. Similar findings were obtained by excluding weakly symptomatic *SOD1* carriers from the LTS group. Using a multilogistic regression, only current age, and not genotype, sex, and plasma NfL, was able to predict the status of long‐term survivor (current age: OR = 1.7, 95% CI = 1.37–2.37, *p* < 0.0001), suggesting that age is the main determinant of penetrance.

**FIGURE 4:**
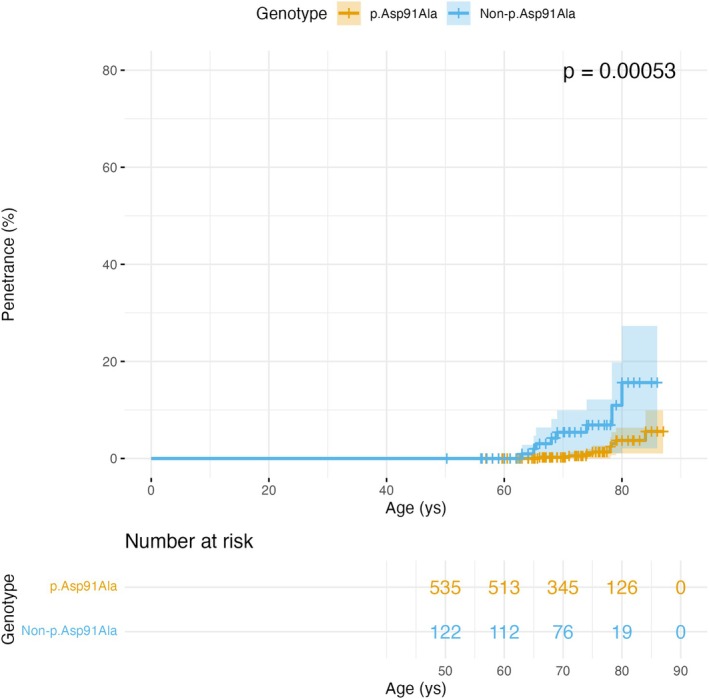
Age‐dependent penetrance of ALS in SOD1 variant carriers stratified by genotype. Kaplan–Meier penetrance curves comparing p.Asp91Ala (*orange*, n = 535) and non‐p.Asp91Ala SOD1 variants (*blue*, n = 122). Penetrance represents the cumulative probability of developing ALS symptoms by a given age. Shaded areas indicate 95% confidence intervals. Tick marks denote censored observations (carriers who remained asymptomatic at last follow‐up). The p.Asp91Ala variant demonstrates significantly lower penetrance compared to other SOD1 variants (*p* = 0.00053, log rank test). Numbers at risk are shown at 10‐year intervals beginning at age 50. Both genotype groups show late‐onset disease with penetrance curves rising predominantly after age 60, although non‐p.Asp91Ala variants exhibit earlier onset and steeper penetrance increase in the 70 to 80 year age range. [Color figure can be viewed at www.annalsofneurology.org]

Cumulative penetrance was significantly higher in non‐p.Asp91Ala carriers than in p.Asp91Ala carriers (log rank test, *p* = 0.00053; Fig 4). In univariate analysis, non‐p.Asp91Ala genotype (HR = 4.65, 95% CI = 1.72–12.13, *p* = 0.001) and baseline NfL (HR = 1.14, 95% CI = 1.05–1.24, *p* = 0.001) were associated with increased risk of symptom development over time. In a multivariate analysis considering also sex, only NfL significantly predicted symptom manifestation (HR = 1.11, 95% CI = 1.01–1.22, *p* = 0.026). The overall model was statistically significant (*p* < 0.001).

The number of symptomatic patients and penetrance estimation across *SOD1* variants is summarized in Supplementary Table S5. To the best of our knowledge, the p.Asp125Val variant was the most penetrant in our cohort (n = 2 symptomatic out of 6, 33.3%), followed by p.Ile114Thr (n = 4 symptomatic out of 32, 12.5%) and the p.Gly73Cys (n = 1 symptomatic out of 25, 4%). Penetrance estimation was not possible for p.Ser69Pro and p.Glu122Gly variants. Considering among symptomatic carriers also those with weak symptoms, penetrance would increase up to 18.5% for p.Ile114Thr (n = 8) and to 24% for p.Gly73Cys (n = 6).

Our estimates of reduced penetrance and longer survival for p.Ile114Thr carriers respect to p.Asp125Val carriers parallels retrospective data collected in the cohort of patients with ALS in Australia,^23^ a country in which British origin is the largest self‐identified ancestry.

With the limitation of clinical data, given the absence of symptomatic subjects, penetrance for the other variants (excluding the p.Asp91Ala) was 0. However, these findings should be interpreted cautiously, given the intrinsic limitations of clinical data collection in the UKB (limited follow‐up time and potential underascertainment of mild disease).

## Discussion

In this work, we identified 122 carriers of pathogenic and likely pathogenic *SOD1* variants among 470,000 participants, indicating a carrier frequency of 1 in 3,826, which is considerably higher than anticipated based on clinical diagnosis alone. This estimate does not include the low penetrant p.Asp91Ala variant, found in heterozygous state in other 535 individuals, and whose individual carrier frequency in the UKB is 1 in 880. Among non‐p.Asp91Ala carriers, 6.6% showed strong or moderate evidence of an MND diagnosis, and elevated baseline plasma NfL concentrations were significantly associated with symptomatic status. Moreover, we identified a large subset of long‐term survivors, particularly among p.Asp91Ala carriers, and we tried to investigate factors possibly influencing penetrance.

Our comprehensive analysis of *SOD1* variants in the UKB cohort reveals a substantial discrepancy between genetic and clinical prevalence of *SOD1*‐ALS, with important implications for screening strategies and therapeutic interventions. This finding is consistent with previous work on the genetic prevalence of short tandem repeat expansions causative for neurological diseases in large genomic cohorts.^20^, ^24^, ^25^ In particular, *C9orf72*‐related ALS was estimated to be over 2 times higher than the prevalence from clinical observation.

The significant deviation of genetic estimates from clinical observation figures likely reflects the complex interplay of several biological and methodological factors. Among all *SOD1* carriers in the UKB, only 2.6% displayed neurological symptoms suggestive of MND. However, ICD‐10 codes do not necessarily inform on symptomatic status, deriving from hospital records and death registries or being self‐reported, potentially missing cases where diagnostic uncertainty persists or where patients have not yet progressed to advanced stages warranting formal medical coding. In addition, it should be acknowledged that, as ICD‐10 codes in UKB are updated periodically, some participants who have developed clinical features or received a diagnosis compatible with MND may not yet be recorded in the biobank data. The inherent limitations of ICD‐10 coding in capturing subtle neurological presentations may contribute to substantial underdiagnosis.

Moreover, to avoid overestimation of symptomatic patients, we deliberately excluded those with mild neurological manifestations (weak symptoms), such as muscle cramps, fasciculations, and nonspecific motor symptoms that, while potentially representing early presentations of MND, especially in *SOD1* carriers, necessitate clinical evaluation and instrumental tests to meet the criteria for ALS. Additionally, the slowly progressive and often insidious nature of *SOD1*‐associated ALS, reported for some genetic backgrounds,^3^, ^26^, ^27^, ^28^, ^29^, ^30^, ^31^ may result in prolonged subclinical phases where symptoms either remain below the threshold for medical attention or are attributed to aging or other benign conditions. Phenotypic heterogeneity also likely contributes to underdiagnosis, as some *SOD1*‐ALS cases present with atypical features that may not prompt neurological evaluation or accurate diagnosis.^32^

On the other hand, we believe that incomplete age‐related penetrance contributes significantly to the observed discordance between genetic and clinical prevalence in population cohorts. Symptomatic carriers had a significantly older age at recruitment compared to asymptomatic ones, suggesting also the presence of an age‐related reduced penetrance. Thus, we hypothesize that at least part of young individuals may have not developed symptoms or signs of MND yet. In support of this, we investigated the subset of asymptomatic carriers aged over 70 years in our cohort, referred to as LTS. Compared with symptomatic patients, they showed a significantly higher proportion of p.Asp91Ala carriers and lower plasma NfL levels at baseline. Age, but not genotype and NfL, was the only significant predictor of long‐term survivor status.

In addition, *SOD1* variants, whereas typically transmitted as autosomal dominant traits, frequently exhibited reduced penetrance resulting in apparently recessive or sporadic presentations.^3^, ^33^ Despite comprehensive penetrance data remaining more limited in the literature and deriving from retrospective studies on familiar cases, the broader pattern of variable expressivity and incomplete penetrance appears to be a shared characteristic across multiple *SOD1* variants.

In our cohort, the p.Ile114Thr variant represents the second most common *SOD1* variant, confirming findings from previous studies.^34^, ^35^ A recent epidemiological study reported this variant as the most common *SOD1* variant in Scotland, causing 2.7% of total cases and up to 18% of familial ones.^36^ According to case series, the p.Ile114Thr variant shows age‐dependent but incomplete penetrance: approximately 50% (8 out of 15) of carriers develop ALS by 60 years of age, but penetrance rises to approximately 88 to 95% before the eighth decade of life.^37^, ^38^ The median age at symptom onset was typically in the fifth to sixth decade, with most cohorts reporting a range between 45 and 55 years.^37^, ^38^

In contrast to previous reports, our cohort shows a relatively high proportion of asymptomatic carriers, likely reflecting age‐dependent penetrance and competing mortality, with several individuals still in their 50s and 60s and 2 non‐ALS deaths before age 80 years that may have censored disease manifestation.

The p.Ser69Pro has been demonstrated so far as causative for early‐onset ALS only in homozygosis. In this study, the same variant was identified in heterozygosis in a single subject recruited at the age of 56 years, who died of MND. Thus, this finding confirms the association between monoallelic defects, initially described as recessive alleles, and late‐onset clinical manifestations.

The p.Asp91Ala variant demonstrates complex inheritance patterns, functioning as either recessive or dominant, with reduced penetrance.^39^ In our cohort, only 1.75% of p.Asp91Ala *SOD1* carriers were symptomatic for MND, with 6.1% showing mild symptoms, compared with 6.6% observed in non‐p.Asp91Ala *SOD1* carriers. The significantly higher cumulative penetrance found in non‐p.Asp91Ala carriers in the UKB cohort further supports the genotype contribution in phenotypic expression.

A recent study used an approach to estimate penetrance in autosomal dominant traits from population‐scale genetic data, suggesting a maximal penetrance of 1 for p.Ala5Val variant, and 0.64 and 0, for p.Ile114Thr and p.Asp91Ala variants, respectively, the latter reaching polymorphic frequency in some populations.^40^ A recent genome‐wide association study (GWAS) reported that the p.Asp91Ala variant might represent a strong genetic risk factor^41^ for ALS. Next‐generation sequencing (NGS) analyses on a small cohort of patients carrying monoallelic and biallelic p.Asp91Ala variants suggested an individual oligogenic background underlying both sporadic and familial cases.^42^

In the UKB, we found a carrier frequency of 1 in 880 (0.0011) for the p.Asp91Ala variant, which is 10 times lower than the one found in Finnish Europeans, and almost doubled compared to carrier frequency in non‐Finnish Europeans (gnomAD MAF 0.0006136).

The 4‐fold difference between genetic prevalence (1.04 per 100,000) and clinical prevalence (0.27 per 100,000) has substantial clinical implications. This gap suggests that many individuals who could benefit from *SOD1*‐directed therapies remain undiagnosed. The recent approval of tofersen for patients with *SOD1*‐ALS provides unprecedent opportunities for intervention, potentially modifying the disease course if administered early. Furthermore, emerging evidence from longitudinal studies of pre‐symptomatic carriers indicates that biomarkers like plasma NfL can identify individuals approaching phenoconversion, creating a window for preventive treatment before irreversible neurodegeneration occurs.^13^

Our finding that the heterozygous p.Asp91Ala variant may cause phenotypes compatible with MND in a limited portion of carriers supports the contribution of this variant also in the heterozygous state, as previously observed.^3^ However, the low rate of symptomatic individuals among p.Asp91Ala carriers acknowledges the role of p.Asp91Ala as a low‐penetrance allele requiring additional genetic or environmental factors to manifest disease, consistent with conclusions from previous family studies and genome‐association studies.^41^, ^43^ Nevertheless, the reduction in serum NfL levels following tofersen administration,^37^ despite to a lower extent compared with homozygous carriers, seems to support the eligibility of symptomatic heterozygous p.Asp91Ala carriers for this molecular therapy. However, the short observation period, slow disease progression, and limited cohort size preclude drawing firm conclusions about clinical treatment response in this study.^37^

Although experience with tofersen in *SOD1*‐ALS is growing by the day,^44^, ^45^, ^46^, ^47^, ^48^ still much is unknown about the exact genotype‐to‐therapy response. Questions regarding variable disease progression after treatment and effective targeting of upper motor neuron signs and cognitive symptoms remain open. In addition, long‐term efficacy data are still missing.

Whereas the availability of Tofersen represents a paradigm shift that may make presymptomatic genetic testing for *SOD1* variants increasingly attractive, the current therapeutic landscape presents significant ethical and practical challenges that warrant careful consideration. The ATLAS study on tofersen administration in presymptomatic *SOD1* carriers (NCT04856982) includes only rapidly progressive variants (or *SOD1* mutation that is approved for inclusion by an external mutation adjudication committee). Future research should focus on identifying additional genetic and environmental modifiers affecting *SOD1* variant penetrance, detecting reliable biomarkers of treatment response, and developing improved predictive models of phenoconversion risk. Implementation studies examining the clinical utility and cost‐effectiveness of broader genetic screening programs would also provide valuable guidance for clinical practice and to support policy decisions in national health services.

Several important limitations warrant consideration when interpreting our findings. The UKB cohort comprises individuals aged 40 to 69 years at recruitment, which may systematically exclude both early‐ and late‐onset cases, thus potentially biasing our prevalence estimates. Furthermore, the reliance on ICD‐10 codes for clinical ascertainment introduces inherent constraints, as these codes are recorded and updated only upon hospital admission regardless of symptom presence, thereby failing to capture the complete clinical trajectory of affected individuals at any given timepoint. The finite follow‐up duration may prove insufficient for detecting late‐onset disease manifestations, particularly given the variable age of onset characteristic of *SOD1*‐ALS. Additionally, the absence of family history data regarding neuromuscular disorders and the lack of systematic instrumental investigations to identify preclinical or subclinical motor neuron dysfunction or abnormal electromyographic activity represent significant gaps in our phenotypic characterization. From a genetic perspective, our analysis focused primarily on previously reported pathogenic single nucleotide variants in *SOD1* coding regions, deliberately excluding variants lacking conclusive pathogenicity evidence as well as structural and non‐coding variants that may contribute to disease burden. We identified several novel *SOD1* variants of uncertain significance that were not incorporated into our genetic prevalence calculations due to insufficient pathogenicity evidence, although we acknowledge that their inclusion would substantially increase prevalence estimates. However, this conservative approach is counterbalanced by emerging evidence from large‐scale genomic databases, including a recent study demonstrating elevated *SOD1* variant frequencies in gnomAD, which suggests reduced penetrance of *SOD1*‐ALS in the general population and tempers the anticipated clinical impact of variants identified through population‐based genomic screening.^49^ Our modeling approach applied a uniform penetrance estimate of 54% across all *SOD1* variants based on prior literature, yet this fails to capture the substantial inter‐variant heterogeneity in both penetrance and age of onset that characterizes *SOD1*‐associated disease.^5^ Finally, whereas the UKB includes participants from diverse ancestries, the cohort remains predominantly composed of individuals of European descent, which may inadequately represent the genetic architecture and variant spectrum of *SOD1*‐ALS across global populations. From a methodological standpoint, our cross‐sectional analytical approach utilized current age as a proxy for age at symptom onset, which may introduce temporal bias in our estimates. Additionally, the limited sample size of symptomatic participants (n = 17) constrained statistical power for certain analyses and may have contributed to overfitting in our predictive modeling, warranting cautious interpretation of these results.

In conclusion, our study reveals that *SOD1*‐related ALS is significantly more prevalent at the genetic level than previously recognized through clinical diagnosis alone. Given that *SOD1*‐ALS is currently the only genetic form of ALS with an approved molecular therapy,^50^ and that early intervention may be critical for treatment efficacy, these results underscore the importance of identifying genetic and environmental factors, as well as biological indicators, able to influence disease penetrance and phenoconversion risk in presymptomatic carriers and to predict treatment response in patients.

## Author Contributions

DG, AT and DR contributed to the conception and design of the study; DG, CV, MZ, VI and DR contributed to the acquisition and analysis of data; DG, CV, MZ, HH, PF, SC and GPC contributed to drafting the text or preparing the figures.

## Potential Conflicts of Interest

The authors report no conflicts of interest.

## Supporting information


**Supplementary Table S1.** Disease modeling of *SOD1*‐ALS.
**Supplementary Table S2**. Disease modeling of *SOD1*‐ALS including p.Asp91Ala carriers.
**Supplementary Table S3**. ICD‐10 codes compatible with motor neuron disease with strong, moderate and weak evidence.
**Supplementary Table S4**. *SOD1* coding variants in UK Biobank not reported in the literature and with no conclusive demonstration of pathogenicity.
**Supplementary Table S5**. Clinical status and penetrance estimation in carriers of *SOD1* pathogenic and likely pathogenic variants.

## Data Availability

All data produced in the present study are available upon reasonable request to the corresponding authors.
