# Supplementary material for: High Prevalence of SOD1 Pathogenic Variants in the UK Biobank: Implications for Early Intervention in Amyotrophic Lateral Sclerosis
Source: Ann Neurol. 2026 Mar 19;99(6):1502–15. doi: 10.1002/ana.78195 (PMC13206312; doi:10.1002/ana.78195)
Supplement: Supplementary file 1 — Supplementary Table S1. Disease modeling of SOD1‐ALS. Supplementary Table S2. Disease modeling of SOD1‐ALS including p.Asp91Ala carriers. Supplementary Table S3. ICD‐10 codes compatible with motor neuron disease with strong, moderate and weak evidence. Supplementary Table S4. SOD1 coding variants in UK Biobank not reported in the literature and with no conclusive demonstration of pathogenicity. Supplementary Table S5. Clinical status and penetrance estimation in carriers of SOD1 pathogenic and likely pathogenic variants. [file ANA-99-1502-s001.docx]

**Supplementary table 1. Disease modelling of *SOD1*-ALS.**

| **Age** | **UK population (ONS data)** | **Onset count** | **Standardized onset count** | **Carrier frequency times standardized onset count** | **Penetrance-corrected estimated UK population** | **Mortality-corrected prevalence estimate** |
| --- | --- | --- | --- | --- | --- | --- |
| 0 | 701897 | 0 | 0 | 0 | 0 | 0 |
| 1 | 730219 | 0 | 0 | 0 | 0 | 0 |
| 2 | 759354 | 0 | 0 | 0 | 0 | 0 |
| 3 | 783321 | 0 | 0 | 0 | 0 | 0 |
| 4 | 807539 | 0 | 0 | 0 | 0 | 0 |
| 5 | 806745 | 0 | 0 | 0 | 0 | 0 |
| 6 | 813073 | 0 | 0 | 0 | 0 | 0 |
| 7 | 830700 | 0 | 0 | 0 | 0 | 0 |
| 8 | 855000 | 0 | 0 | 0 | 0 | 0 |
| 9 | 841895 | 0 | 0 | 0 | 0 | 0 |
| 10 | 826080 | 0 | 0 | 0 | 0 | 0 |
| 11 | 817287 | 0 | 0 | 0 | 0 | 0 |
| 12 | 823732 | 0 | 0 | 0 | 0 | 0 |
| 13 | 796700 | 3 | 0,005016722 | 1,29164E-06 | 0,555685963 | 0,555685963 |
| 14 | 781315 | 0 | 0 | 0 | 0 | 0,555685963 |
| 15 | 752712 | 0 | 0 | 0 | 0 | 0,555685963 |
| 16 | 740693 | 0 | 0 | 0 | 0 | 0,555685963 |
| 17 | 722928 | 1 | 0,001672241 | 4,30546E-07 | 0,168077044 | 0,723763007 |
| 18 | 717252 | 0 | 0 | 0 | 0 | 0,723763007 |
| 19 | 750095 | 1 | 0,001672241 | 4,30546E-07 | 0,174393231 | 0,342470275 |
| 20 | 778930 | 0 | 0 | 0 | 0 | 0,342470275 |
| 21 | 810303 | 3 | 0,005016722 | 1,29164E-06 | 0,565173846 | 0,907644121 |
| 22 | 827261 | 3 | 0,005016722 | 1,29164E-06 | 0,577001789 | 1,48464591 |
| 23 | 856602 | 1 | 0,001672241 | 4,30546E-07 | 0,199155561 | 1,515724428 |
| 24 | 860062 | 3 | 0,005016722 | 1,29164E-06 | 0,599879981 | 2,115604409 |
| 25 | 858738 | 2 | 0,003344482 | 8,61092E-07 | 0,399304341 | 2,340515519 |
| 26 | 884489 | 2 | 0,003344482 | 8,61092E-07 | 0,411278291 | 2,75179381 |
| 27 | 887131 | 2 | 0,003344482 | 8,61092E-07 | 0,412506794 | 2,599126758 |
| 28 | 914604 | 6 | 0,010033445 | 2,58328E-06 | 1,27584437 | 3,297969338 |
| 29 | 931668 | 6 | 0,010033445 | 2,58328E-06 | 1,299648123 | 4,3984619 |
| 30 | 914186 | 9 | 0,015050167 | 3,87491E-06 | 1,912891909 | 5,711473828 |
| 31 | 905920 | 7 | 0,011705686 | 3,01382E-06 | 1,4743522 | 6,786521687 |
| 32 | 913691 | 7 | 0,011705686 | 3,01382E-06 | 1,486999223 | 7,86224262 |
| 33 | 891015 | 5 | 0,008361204 | 2,15273E-06 | 1,035782035 | 8,485517861 |
| 34 | 897163 | 12 | 0,02006689 | 5,16655E-06 | 2,503029425 | 9,712702916 |
| 35 | 894424 | 11 | 0,018394649 | 4,73601E-06 | 2,287438802 | 10,7004936 |
| 36 | 872466 | 9 | 0,015050167 | 3,87491E-06 | 1,825594739 | 10,61319643 |
| 37 | 878205 | 14 | 0,023411371 | 6,02764E-06 | 2,858494071 | 11,9973383 |
| 38 | 876420 | 16 | 0,026755853 | 6,88874E-06 | 3,260210313 | 13,77054939 |
| 39 | 882585 | 16 | 0,026755853 | 6,88874E-06 | 3,283143606 | 16,01791096 |
| 40 | 882352 | 14 | 0,023411371 | 6,02764E-06 | 2,871992257 | 16,38687379 |
| 41 | 846976 | 12 | 0,02006689 | 5,16655E-06 | 2,363010791 | 16,46244578 |
| 42 | 790568 | 18 | 0,030100334 | 7,74983E-06 | 3,308453926 | 17,94530496 |
| 43 | 778286 | 17 | 0,028428094 | 7,31928E-06 | 3,076107399 | 18,16291829 |
| 44 | 793361 | 21 | 0,035117057 | 9,04147E-06 | 3,873499435 | 18,77620741 |
| 45 | 807777 | 23 | 0,038461538 | 9,90256E-06 | 4,319491999 | 19,81255581 |
| 46 | 821621 | 19 | 0,031772575 | 8,18037E-06 | 3,629430517 | 20,56999407 |
| 47 | 857254 | 18 | 0,030100334 | 7,74983E-06 | 3,587528666 | 21,79451194 |
| 48 | 894159 | 21 | 0,035117057 | 9,04147E-06 | 4,365634788 | 22,8516928 |
| 49 | 923156 | 27 | 0,045150502 | 1,16247E-05 | 5,794983656 | 25,57056906 |
| 50 | 901680 | 21 | 0,035117057 | 9,04147E-06 | 4,402355259 | 26,09942489 |
| 51 | 923655 | 24 | 0,040133779 | 1,03331E-05 | 5,153880942 | 26,93381383 |
| 52 | 923357 | 10 | 0,016722408 | 4,30546E-06 | 2,146757558 | 25,45114087 |
| 53 | 934714 | 18 | 0,030100334 | 7,74983E-06 | 3,911691598 | 25,7753038 |
| 54 | 932611 | 16 | 0,026755853 | 6,88874E-06 | 3,469236211 | 24,87890522 |
| 55 | 938738 | 15 | 0,025083612 | 6,45819E-06 | 3,273776388 | 22,35769796 |
| 56 | 928163 | 11 | 0,018394649 | 4,73601E-06 | 2,373724387 | 20,32906708 |
| 57 | 906643 | 11 | 0,018394649 | 4,73601E-06 | 2,318688204 | 17,49387435 |
| 58 | 884567 | 9 | 0,015050167 | 3,87491E-06 | 1,850915522 | 17,19803231 |
| 59 | 852740 | 19 | 0,031772575 | 8,18037E-06 | 3,76689566 | 17,05323637 |
| 60 | 816209 | 14 | 0,023411371 | 6,02764E-06 | 2,656701552 | 16,24070171 |
| 61 | 796532 | 10 | 0,016722408 | 4,30546E-06 | 1,851895953 | 14,81882128 |
| 62 | 777771 | 5 | 0,008361204 | 2,15273E-06 | 0,904138796 | 13,34923569 |
| 63 | 747241 | 6 | 0,010033445 | 2,58328E-06 | 1,042378147 | 12,07292563 |
| 64 | 718065 | 10 | 0,016722408 | 4,30546E-06 | 1,669464211 | 11,89147432 |
| 65 | 689198 | 7 | 0,011705686 | 3,01382E-06 | 1,121644944 | 9,246223603 |
| 66 | 687334 | 9 | 0,015050167 | 3,87491E-06 | 1,438214594 | 8,027736645 |
| 67 | 674764 | 5 | 0,008361204 | 2,15273E-06 | 0,784395806 | 6,960236499 |
| 68 | 651687 | 5 | 0,008361204 | 2,15273E-06 | 0,757569387 | 6,81366709 |
| 69 | 652398 | 10 | 0,016722408 | 4,30546E-06 | 1,516791812 | 7,288080755 |
| 70 | 660817 | 7 | 0,011705686 | 3,01382E-06 | 1,075455888 | 6,694072432 |
| 71 | 672642 | 4 | 0,006688963 | 1,72218E-06 | 0,625543229 | 6,197970716 |
| 72 | 702415 | 6 | 0,010033445 | 2,58328E-06 | 0,979847259 | 5,739603381 |
| 73 | 753009 | 1 | 0,001672241 | 4,30546E-07 | 0,175070721 | 5,130278296 |
| 74 | 575023 | 2 | 0,003344482 | 8,61092E-07 | 0,267379783 | 4,640088692 |
| 75 | 549939 | 1 | 0,001672241 | 4,30546E-07 | 0,12785799 | 3,251154871 |
| 76 | 540477 | 2 | 0,003344482 | 8,61092E-07 | 0,251316248 | 2,427015231 |
| 77 | 494925 | 3 | 0,005016722 | 1,29164E-06 | 0,345202555 | 2,146674557 |
| 78 | 435050 | 4 | 0,006688963 | 1,72218E-06 | 0,404587554 | 1,571414852 |
| 79 | 383368 | 0 | 0 | 0 | 0 | 1,39634413 |
| 80 | 386890 | 1 | 0,001672241 | 4,30546E-07 | 0,089949936 | 1,218914283 |
| 81 | 373288 | 1 | 0,001672241 | 4,30546E-07 | 0,086787541 | 1,177843834 |
| 82 | 350944 | 0 | 0 | 0 | 0 | 0,926527586 |
| 83 | 322102 | 0 | 0 | 0 | 0 | 0,581325031 |
| 84 | 292999 | 0 | 0 | 0 | 0 | 0,176737477 |
| 85 | 264997 | 2 | 0,003344482 | 8,61092E-07 | 0,12322088 | 0,299958358 |
| 86 | 231822 | 0 | 0 | 0 | 0 | 0,210008421 |
| 87 | 206428 | 0 | 0 | 0 | 0 | 0,12322088 |
| 88 | 185189 | 0 | 0 | 0 | 0 | 0,12322088 |
| 89 | 161430 | 0 | 0 | 0 | 0 | 0,12322088 |
| 90 | 609503 | 0 | 0 | 0 | 0 | 0,12322088 |
|  | **67081234** | **598** |  |  |  | **700,3161337** |

**carrier frequency in UK Biobank: 1/3884**

predicted population prevalence (1 in …): 95787.07

**predicted population prevalence out of 100.000: 1.04**

**Supplementary table 2. Disease modelling of *SOD1*-ALS including p.Asp91Ala carriers.**

| **Age** | **UK population (ONS data)** | **Onset count** | **Standardized onset count** | **Carrier frequency times standardized onset count** | **Penetrance-corrected estimated UK population** | **Mortality-corrected prevalence estimate** |
| --- | --- | --- | --- | --- | --- | --- |
| 0 | 701897 | 0 | 0 | 0 | 0 | 0 |
| 1 | 730219 | 0 | 0 | 0 | 0 | 0 |
| 2 | 759354 | 0 | 0 | 0 | 0 | 0 |
| 3 | 783321 | 0 | 0 | 0 | 0 | 0 |
| 4 | 807539 | 0 | 0 | 0 | 0 | 0 |
| 5 | 806745 | 0 | 0 | 0 | 0 | 0 |
| 6 | 813073 | 0 | 0 | 0 | 0 | 0 |
| 7 | 830700 | 0 | 0 | 0 | 0 | 0 |
| 8 | 855000 | 0 | 0 | 0 | 0 | 0 |
| 9 | 841895 | 0 | 0 | 0 | 0 | 0 |
| 10 | 826080 | 0 | 0 | 0 | 0 | 0 |
| 11 | 817287 | 0 | 0 | 0 | 0 | 0 |
| 12 | 823732 | 0 | 0 | 0 | 0 | 0 |
| 13 | 796700 | 3 | 0,004431315 | 6,17175E-06 | 2,655196817 | 2,655196817 |
| 14 | 781315 | 0 | 0 | 0 | 0 | 2,655196817 |
| 15 | 752712 | 0 | 0 | 0 | 0 | 2,655196817 |
| 16 | 740693 | 0 | 0 | 0 | 0 | 2,655196817 |
| 17 | 722928 | 1 | 0,001477105 | 2,05725E-06 | 0,803111219 | 3,458308036 |
| 18 | 717252 | 0 | 0 | 0 | 0 | 3,458308036 |
| 19 | 750095 | 1 | 0,001477105 | 2,05725E-06 | 0,833291434 | 1,636402653 |
| 20 | 778930 | 0 | 0 | 0 | 0 | 1,636402653 |
| 21 | 810303 | 3 | 0,004431315 | 6,17175E-06 | 2,700532128 | 4,336934781 |
| 22 | 827261 | 3 | 0,004431315 | 6,17175E-06 | 2,757048794 | 7,093983575 |
| 23 | 856602 | 1 | 0,001477105 | 2,05725E-06 | 0,951611608 | 7,242483964 |
| 24 | 860062 | 3 | 0,004431315 | 6,17175E-06 | 2,866366116 | 10,10885008 |
| 25 | 858738 | 2 | 0,00295421 | 4,1145E-06 | 1,907969043 | 11,18352769 |
| 26 | 884489 | 2 | 0,00295421 | 4,1145E-06 | 1,965183363 | 13,14871105 |
| 27 | 887131 | 2 | 0,00295421 | 4,1145E-06 | 1,971053435 | 12,41923236 |
| 28 | 914604 | 6 | 0,008862629 | 1,23435E-05 | 6,096281234 | 15,7584648 |
| 29 | 931668 | 8 | 0,011816839 | 1,6458E-05 | 8,280028143 | 23,08688133 |
| 30 | 914186 | 9 | 0,013293944 | 1,85152E-05 | 9,140242591 | 29,36075781 |
| 31 | 905920 | 8 | 0,011816839 | 1,6458E-05 | 8,051197525 | 35,50398629 |
| 32 | 913691 | 9 | 0,013293944 | 1,85152E-05 | 9,135293467 | 42,67409639 |
| 33 | 891015 | 7 | 0,010339734 | 1,44007E-05 | 6,928890567 | 47,63193353 |
| 34 | 897163 | 14 | 0,020679468 | 2,88015E-05 | 13,95339977 | 55,48905206 |
| 35 | 894424 | 13 | 0,019202363 | 2,67442E-05 | 12,91717202 | 60,12619594 |
| 36 | 872466 | 10 | 0,014771049 | 2,05725E-05 | 9,692351559 | 60,67830491 |
| 37 | 878205 | 15 | 0,022156573 | 3,08587E-05 | 14,63416042 | 67,2612678 |
| 38 | 876420 | 16 | 0,023633678 | 3,2916E-05 | 15,57804339 | 73,70401773 |
| 39 | 882585 | 18 | 0,026587888 | 3,70305E-05 | 17,648577 | 84,42370416 |
| 40 | 882352 | 15 | 0,022156573 | 3,08587E-05 | 14,70326485 | 85,17356924 |
| 41 | 846976 | 13 | 0,019202363 | 2,67442E-05 | 12,23193328 | 84,4883305 |
| 42 | 790568 | 20 | 0,029542097 | 4,1145E-05 | 17,56506956 | 92,3610485 |
| 43 | 778286 | 18 | 0,026587888 | 3,70305E-05 | 15,56296606 | 93,28985414 |
| 44 | 793361 | 24 | 0,035450517 | 4,9374E-05 | 21,15255029 | 98,86436104 |
| 45 | 807777 | 25 | 0,036927622 | 5,14312E-05 | 22,43428015 | 103,6500642 |
| 46 | 821621 | 24 | 0,035450517 | 4,9374E-05 | 21,90601696 | 110,8528163 |
| 47 | 857254 | 20 | 0,029542097 | 4,1145E-05 | 19,04671848 | 117,6676015 |
| 48 | 894159 | 23 | 0,033973412 | 4,73167E-05 | 22,84668717 | 122,9492191 |
| 49 | 923156 | 32 | 0,047267356 | 6,5832E-05 | 32,81751723 | 140,2037703 |
| 50 | 901680 | 23 | 0,033973412 | 4,73167E-05 | 23,0388565 | 142,0900765 |
| 51 | 923655 | 26 | 0,038404727 | 5,34885E-05 | 26,67864575 | 146,3344421 |
| 52 | 923357 | 11 | 0,016248154 | 2,26297E-05 | 11,28347778 | 135,7119029 |
| 53 | 934714 | 20 | 0,029542097 | 4,1145E-05 | 20,76774727 | 137,4329317 |
| 54 | 932611 | 18 | 0,026587888 | 3,70305E-05 | 18,64891999 | 133,2351645 |
| 55 | 938738 | 19 | 0,028064993 | 3,90877E-05 | 19,81429599 | 120,2319433 |
| 56 | 928163 | 13 | 0,019202363 | 2,67442E-05 | 13,40442691 | 110,5975137 |
| 57 | 906643 | 11 | 0,016248154 | 2,26297E-05 | 11,0792317 | 94,99809964 |
| 58 | 884567 | 10 | 0,014771049 | 2,05725E-05 | 9,826783326 | 93,54140518 |
| 59 | 852740 | 21 | 0,031019202 | 4,32022E-05 | 19,89374637 | 92,66740429 |
| 60 | 816209 | 15 | 0,022156573 | 3,08587E-05 | 13,60107656 | 87,61956086 |
| 61 | 796532 | 11 | 0,016248154 | 2,26297E-05 | 9,733668692 | 77,53893356 |
| 62 | 777771 | 8 | 0,011816839 | 1,6458E-05 | 6,912296836 | 71,04680349 |
| 63 | 747241 | 10 | 0,014771049 | 2,05725E-05 | 8,30120884 | 68,26878063 |
| 64 | 718065 | 11 | 0,016248154 | 2,26297E-05 | 8,774797258 | 67,21679456 |
| 65 | 689198 | 7 | 0,010339734 | 1,44007E-05 | 5,359480503 | 52,68252869 |
| 66 | 687334 | 12 | 0,017725258 | 2,4687E-05 | 9,162831927 | 48,24428406 |
| 67 | 674764 | 7 | 0,010339734 | 1,44007E-05 | 5,247235921 | 43,75785129 |
| 68 | 651687 | 6 | 0,008862629 | 1,23435E-05 | 4,343811342 | 41,18936579 |
| 69 | 652398 | 10 | 0,014771049 | 2,05725E-05 | 7,247584172 | 40,13574112 |
| 70 | 660817 | 8 | 0,011816839 | 1,6458E-05 | 5,872889653 | 37,23383352 |
| 71 | 672642 | 4 | 0,005908419 | 8,229E-06 | 2,988991084 | 34,8633441 |
| 72 | 702415 | 6 | 0,008862629 | 1,23435E-05 | 4,681938176 | 30,38245035 |
| 73 | 753009 | 2 | 0,00295421 | 4,1145E-06 | 1,673057278 | 26,80827171 |
| 74 | 575023 | 2 | 0,00295421 | 4,1145E-06 | 1,277602811 | 23,74206317 |
| 75 | 549939 | 2 | 0,00295421 | 4,1145E-06 | 1,221870451 | 17,71634945 |
| 76 | 540477 | 2 | 0,00295421 | 4,1145E-06 | 1,200847504 | 13,0443073 |
| 77 | 494925 | 3 | 0,004431315 | 6,17175E-06 | 1,649458121 | 11,70477434 |
| 78 | 435050 | 5 | 0,007385524 | 1,02862E-05 | 2,41651683 | 9,439352995 |
| 79 | 383368 | 1 | 0,001477105 | 2,05725E-06 | 0,425889081 | 8,192184799 |
| 80 | 386890 | 1 | 0,001477105 | 2,05725E-06 | 0,429801722 | 7,34438371 |
| 81 | 373288 | 1 | 0,001477105 | 2,05725E-06 | 0,414691063 | 6,537204322 |
| 82 | 350944 | 0 | 0 | 0 | 0 | 5,336356818 |
| 83 | 322102 | 0 | 0 | 0 | 0 | 3,686898697 |
| 84 | 292999 | 0 | 0 | 0 | 0 | 1,270381867 |
| 85 | 264997 | 2 | 0,00295421 | 4,1145E-06 | 0,588778035 | 1,43327082 |
| 86 | 231822 | 1 | 0,001477105 | 2,05725E-06 | 0,257534428 | 1,261003526 |
| 87 | 206428 | 0 | 0 | 0 | 0 | 0,846312463 |
| 88 | 185189 | 0 | 0 | 0 | 0 | 0,846312463 |
| 89 | 161430 | 0 | 0 | 0 | 0 | 0,846312463 |
| 90 | 609503 | 0 | 0 | 0 | 0 | 0,846312463 |
|  | **67081234** | **677** |  |  |  | **3779,466439** |

**carrier frequency (including the p.Asp91Ala) in UK Biobank: 1/718**

predicted population prevalence (1 in …) 17748.9

**predicted population prevalence out of 100.000 5.63**

**Supplementary table 3. ICD-10 codes compatible with motor neuron disease with strong. moderate and weak evidence.**

| **STRONG** |
| --- |
| G12.1 Other inherited spinal muscular atrophy |
| G12.2 Motor neuron disease |
| G12.8 Other spinal muscular atrophies and related syndromes |
| **MODERATE** |
| G31.8 Other specified degenerative diseases of nervous system |
| G31.9 Degenerative disease of the nervous system, unspecified |
| G32.8 Other specified degenerative disorders of nervous system in diseases classified elsewhere |
| G62.9 Polyneuropathy. unspecified |
| G62.8 Other specified polyneuropathies |
| **WEAK** |
| G31.0 Circumscribed brain atrophy |
| G31.1 Senile degeneration of brain not elsewhere classified |
| G60.3 Idiopathic progressive neuropathy |
| G60.8 Other hereditary and idiopathic neuropathies |
| G60.9 Hereditary and idiopathic neuropathy. unspecified |
| G63.6 Polyneuropathy in other musculoskeletal disorders |
| G63.8 Polyneuropathy in other diseases classified elsewhere |
| G64 Other disorders of peripheral nervous system |
| R25.2 Cramp and spasm |
| R25.3 Fasciculation |
| R25.8 Other and unspecified abnormal involuntary movements |
| R26.1 Paralytic gait |
| R26.2 Difficulty in walking. not elsewhere classified |
| R26.3 Immobility |
| R26.8 Other and unspecified abnormalities of gait and mobility |
| R29.2 Abnormal reflex |
| R29.6 Tendency to fall. not elsewhere classified |
| R29.8 Other and unspecified symptoms and signs involving the nervous and musculoskeletal systems |
| R47.0 Dysphasia and aphasia |
| R47.1 Dysarthria and anarthria |
| R47.8 Other and unspecified speech disturbances |
| R49.0 Dysphonia |
| R49.1 Aphonia |
| R49.8 Other and unspecified voice disturbances |

**Supplementary table 4. *SOD1* coding variants in UK Biobank not reported in the literature and with no conclusive demonstration of pathogenicity.**

| **Nucleotide Change** | **Protein Change** | **Exon** | **UKB frequency** | **ACMG Criteria** | **P/LP/VUS** | **Variant Type** | **Databases** |
| --- | --- | --- | --- | --- | --- | --- | --- |
| c.8C>G | p.Thr3Arg | 1 | 2 | PM1. PP2. PM2 | VUS | Nonsynonymous SNV |  |
| c.22G>A | p.Val8Met | 1 | 1 | PM1. PP2. PM2. PM5. PP3 | LP | Nonsynonymous SNV |  |
| c.34G>A | p.Asp12Asn | 1 | 1 | PM1. PP2. PM2. PM5 | LP | Nonsynonymous SNV |  |
| c.36C>A | p.Asp12Glu | 1 | 2 | PM1. PP2. PM2. PM5 | LP | Nonsynonymous SNV |  |
| c.40C>T | p.Pro14Ser | 1 | 1 | PM1. PP2. PM2 | VUS | Nonsynonymous SNV |  |
| c.47A>G | p.Gln16Arg | 1 | 25 | PM1. PP2. PM2 | VUS | Nonsynonymous SNV |  |
| c.48G>C | p.Gln16His | 1 | 4 | PM1. PP2. PM2 | VUS | Nonsynonymous SNV |  |
| c.50G>T | p.Gly17Val | 1 | 1 | PM1. PP2. PM2. PM5. PP3 | LP | Nonsynonymous SNV |  |
| c.70A>C | p.Lys24Gln | 1 | 1 | PM1. PP2. PM2 | VUS | Nonsynonymous SNV |  |
| c.74A>G | p.Glu25Gly | 2 | 2 | PM2. PP2 | VUS | Nonsynonymous SNV |  |
| c.77G>A | p.Ser26Asn | 2 | 1 | PM2. PP2 | VUS | Nonsynonymous SNV |  |
| c.82G>A | p.Gly28Arg | 2 | 3 | PM2. PM1. PP2 | VUS | Nonsynonymous SNV |  |
| c.86C>G | p.Pro29Arg | 2 | 9 | PM2. PM1. PP2 | VUS | Nonsynonymous SNV |  |
| c.92A>G | p.Lys31Arg | 2 | 2 | PM2. PM1. PP2 | VUS | Nonsynonymous SNV |  |
| c.92A>T | p.Lys31Met | 2 | 1 | PM2. PM1. PP2 | VUS | Nonsynonymous SNV |  |
| c.136T>G | p.Phe46Val | 2 | 1 | PM1. PP2. PM2. PP3. PM5 | LP | Nonsynonymous SNV |  |
| c.138C>G | p.Phe46Leu | 2 | 2 | PM1. PP2. PM2. PM5. PP3 | LP | Nonsynonymous SNV |  |
| c.164C>T | p.Thr55Ile | 2 | 2 | PM1. PP2. PM2. PP3. PM5 | LP | Nonsynonymous SNV |  |
| c.173G>C | p.Cys58Ser | 3 | 1 | PM2. PM1. PP2. PP3 | VUS | Nonsynonymous SNV |  |
| c.181G>A | p.Ala61Thr | 3 | 1 | PM2. PM1. PP2. PP3 | VUS | Nonsynonymous SNV |  |
| c.182C>G | p.Ala61Gly | 3 | 1 | PM2. PM1. PP2. PP3 | VUS | Nonsynonymous SNV |  |
| c.182C>T | p.Ala61Val | 3 | 2 | PM2. PM1. PP2. PP3 | VUS | Nonsynonymous SNV |  |
| c.216C>A | p.His72Gln | 3 | 2 | PM1. PP2. PM2. PM5. PP3 | LP | Nonsynonymous SNV |  |
| c.221G>A | p.Gly74Glu | 3 | 1 | PM1. PP2. PM2. PP3 | LP | Nonsynonymous SNV |  |
| c.229G>A | p.Asp77Asn | 3 | 1 | PM1. PP2. PM2. PM5. PP3 | LP | Nonsynonymous SNV |  |
| c.274A>G | p.Lys92Glu | 4 | 1 | PM1. PP2. PM2 | VUS | Nonsynonymous SNV |  |
| c.276A>T | p.Lys92Asn | 4 | 1 | PM1. PP2. PM2 | VUS | Nonsynonymous SNV |  |
| c.277G>C | p.Asp93His | 4 | 2 | PM1. PP2. PM2 | VUS | Nonsynonymous SNV |  |
| c.278A>G | p.Asp93Gly | 4 | 7 | PM1. PP2. PM2 | VUS | Nonsynonymous SNV |  |
| c.284T>C | p.Val95Ala | 4 | 9 | PM2. PM1. PP2. PP3 | LP | Nonsynonymous SNV | ALSOD. SODCOD |
| c.287C>T | p.Ala96Val | 4 | 5 | PM1. PP2. PM2. PM5. PP3 | LP | Nonsynonymous SNV | SODCOD |
| c.290A>T | p.Asp97Val | 4 | 1 | PM1. PP2. PM2. PM5 | LP | Nonsynonymous SNV |  |
| c.298A>G | p.Ile100Val | 4 | 8 | PM1. PP2. PM2 | VUS | Nonsynonymous SNV | LOVD (VUS). ALSOD (benign/neutral). SODCOD |
| c.299T>C | p.Ile100Thr | 4 | 2 | PM2. PM1. PP2. PP3 | LP | Nonsynonymous SNV |  |
| c.307T>A | p.Ser103Thr | 4 | 8 | PM1. PP2. PM2 | VUS | Nonsynonymous SNV |  |
| c.314T>C | p.Ile105Thr | 4 | 1 | PM1. PP2. PM2. PM5. PP3 | LP | Nonsynonymous SNV |  |
| c.331C>G | p.His111Asp | 4 | 3 | PM1. PP2. PM2 | VUS | Nonsynonymous SNV |  |
| c.335G>C | p.Cys112Ser | 4 | 1 | PM1. PP2. PM2. PM5 | LP | Nonsynonymous SNV |  |
| c.347G>A | p.Arg116His | 4 | 9 | PS4. PM1. PP2. PM2. PM5. PP3 | P | Nonsynonymous SNV |  |
| c.382G>A | p.Gly128Ser | 5 | 1 | PM2. PM1. PP2. PP3 | LP | Nonsynonymous SNV |  |
| c.385A>G | p.Lys129Glu | 5 | 2 | PM2. PM1. PP2. PP3 | LP | Nonsynonymous SNV |  |
| c.388G>A | p.Gly130Ser | 5 | 2 | PM2. PM1. PP2. PP3 | LP | Nonsynonymous SNV |  |
| c.391G>A | p.Gly131Arg | 5 | 1 | PM2. PM1. PP2. PP3 | LP | Nonsynonymous SNV |  |
| c.392G>A | p.Gly131Glu | 5 | 4 | PM2. PM1. PP2. PP3 | LP | Nonsynonymous SNV |  |
| c.407C>T | p.Thr136Ile | 5 | 38 | PM1. PP2. PM2 | VUS | Nonsynonymous SNV |  |
| c.421G>C | p.Ala141Pro | 5 | 1 | PM2. PM1. PP2. PP3 | LP | Nonsynonymous SNV |  |
| c.430C>T | p.Arg144Cys | 5 | 4 | PM2. PM1. PP2. PP3 | LP | Nonsynonymous SNV |  |
| c.431G>A | p.Arg144His | 5 | 2 | PM2. PM1. PP2. PP3 | LP | Nonsynonymous SNV |  |
| c.448A>G | p.Ile150Val | 5 | 1 | PM1. PP2. PM2. PM5. PP5 | LP | Nonsynonymous SNV | SODCOD |
| c.457G>A | p.Ala153Thr | 5 | 2 | PM1. PP2. PM2 | VUS | Nonsynonymous SNV | ProjectMINE (moderate) |
| c.58delA | p.Asn20Ilefs*11 | 1 | 3 | PVS1. PM2 | LP | Frameshift deletion |  |
| c.240_250del | p.His81Leufs*7 | 4 | 3 | PVS1. PM2 | LP | Frameshift deletion |  |
| c.120_121insA | p.Glu41Argfs*10 | 2 | 1 | PVS1. PM2 | LP | Frameshift insertion |  |
| c.421dupG | p.Ala141Glyfs*23 | 5 | 1 | PVS1. PM2 | LP | Frameshift insertion |  |
| c.2T>C | p.Met1? | 1 | 1 | PM2. PVS1 | VUS | Startloss |  |
| c.98G>A | p.Trp33* | 2 | 2 | PVS1. PM2 | LP | Stopgain |  |
| c.323C>G | p.Ser108* | 4 | 1 | PVS1. PM2 | LP | Stopgain |  |
| c.464A>C | p.*155Serext*6 | 5 | 8 | PM2.PM4 | VUS | Stoploss | projectMINE (high) |
| c.465A>T | p.*155Tyrext*6 | 5 | 2 | PM2.PM4 | VUS | Stoploss |  |
| c.72+1G>A |  | 1 | 1 | PVS1. PM2 | LP | Splicing |  |
| c.72+2C>T |  | 1 | 1 | PVS1. PM2 | LP | Splicing |  |
| c.73-2A>G |  | 2 | 2 | PVS1. PM2 | LP | Splicing |  |
| c.357+1insT |  | 4 | 16 | PM2.PP3 | VUS | Splicing |  |
| c.328G>T |  | 4 | 40 | PM1. PP2. PM2 | VUS | Nonsynonymous SNV | ALSOD. SODCOD |
| c.59A>G | p.Asn20Ser | 1 | 81 | PM1. PP2. PM2. BP6 | VUS | Nonsynonymous SNV | LOVD. ALSOD. SODCOD. projectMINE (moderate) |
| c.289G>A | p.Asp97Asn | 4 | 4 | PM1. PP2. PM2. PM5 | LP | Nonsynonymous SNV | ALSOD |

| **Variant** | **Genotype** | **Carriers, N** | **Alive, N** | **Symptomatic, N** | **Long-term survivors, N** | **Penetrance, %** | **Weak symptoms, N** | **Age at recruitment, ys median [IQR]** | **Current age, ys median [IQR]** | **Normalised NfL**  **median [IQR]** |
| --- | --- | --- | --- | --- | --- | --- | --- | --- | --- | --- |
| **p.Asp91Ala** | **p.Asp91Ala** | **535** | **495** | **9** | **321** | **1.7** | 32 | 57 (50-63) | 73 (67-80) | -0.06 (-0.28-0.46) |
| **Non p.Asp91Ala (all)** | **Non p.Asp91Ala** | **122** | **105** | **8** | **68** | **6.6** | 8 | 58 (50.25-62) | 74 (62.25-79) | 0.25 (-0.19-0.77) |
| p.Ile114Thr | Non p.Asp91Ala | 32 | 24 | 4 | 17 | 12.5 | 2 | 58 (51.75-62.25) | 73 (69.25-79) | 0.33 (-0.08 - 8.61) |
| p.Gln23His | Non p.Asp91Ala | 26 | 24 | 0 | 17 | 0 | 0 | 58 (52-63) | 74 (69-78) | 0.71 (0.17-0.84) |
| p.Gly73Cys | Non p.Asp91Ala | 25 | 22 | 1 | 14 | 4 | 5 | 60 (51-63) | 76 (68-81) | 0.23 (0.03- 0.36) |
| p.Asp125Val | Non p.Asp91Ala | 6 | 5 | 2 | 3 | 33.3 | 0 | 61 (59.25-62.75) | 77.15 (76-80) | NA |
| p.Gly73Ser | Non p.Asp91Ala | 5 | 5 | 0 | 2 | 0 | 0 | 49 (41-58) | 64 (57-74) | NA |
| p.Leu9Val | Non p.Asp91Ala | 4 | 3 | 0 | 1 | 0 | 0 | 54.5 (50-57.5) | 65.3 (66-73.75) | NA |
| p.Arg116Cys | Non p.Asp91Ala | 4 | 4 | 0 | 1 | 0 | 0 | 47 (43.25-52.25) | 63.5 (60.25-68.5) | 14.74 |
| p.Thr55Arg | Non p.Asp91Ala | 4 | 4 | 0 | 2 | 0 | 0 | 55.5 (45.5-65) | 72.5 (62-82) | -0.29 |
| p.Asp77Tyr | Non p.Asp91Ala | 3 | 3 | 0 | 3 | 0 | 0 | 60 (58.5-61.5) | 77 ( | 0.28 |
| p.Val6Leu | Non p.Asp91Ala | 3 | 3 | 0 | 2 | 0 | 0 | 60 (53.5-62.5) | 75 (69-78.5) | -0.67 |
| p.Ile19del | Non p.Asp91Ala | 2 | 2 | 0 | 0 | 0 | 0 | 43 (42.5-43.5) | 61 (60.5-61.5) | NA |
| p.Val149Ile | Non p.Asp91Ala | 2 | 2 | 0 | 2 | 0 | 1 | 61 (59-63) | 78 (76.5-79.5) | NA |
| p.Cys112Tyr | Non p.Asp91Ala | 1 | 1 | 0 | 1 | 0 | 0 | 61 | 78 | NA |
| p.Phe21Leu | Non p.Asp91Ala | 1 | 1 | 0 | 1 | 0 | 0 | 58 | 74 | NA |
| p.Glu122Gly | Non p.Asp91Ala | 1 | 0 | 0 | 0 | 0 | 0 | 59 | 67 | NA |
| p.Glu134del | Non p.Asp91Ala | 1 | 1 | 0 | 1 | 0 | 0 | 59 | 75 | NA |
| p.Asn87Ser | Non p.Asp91Ala | 1 | 1 | 0 | 1 | 0 | 0 | 54 | 72 | NA |
| p.Ser69Pro | Non p.Asp91Ala | 1 | 0 | 1 | 0 | 100 | 0 | 56 | 68.8 | NA |

**Supplementary table 5 Clinical status and penetrance estimation in carriers of *SOD1* pathogenic and likely pathogenic variants.**
